# Room Temperature, Hybrid Sodium-Based Flow Batteries with Multi-Electron Transfer Redox Reactions

**DOI:** 10.1038/srep11215

**Published:** 2015-06-11

**Authors:** Jack S. Shamie, Caihong Liu, Leon L. Shaw, Vincent L. Sprenkle

**Affiliations:** 1 Wanger Institute for Sustainable Energy Research; 2 Department of Mechanical, Materials and Aerospace Engineering Illinois Institute of Technology, Chicago, Illinois 60616; 3 Energy Storage and Conversion Energy Materials Pacific Northwest National Laboratory, Richland, WA 99352

## Abstract

We introduce a new concept of hybrid Na-based flow batteries (HNFBs) with a molten Na alloy anode in conjunction with a flowing catholyte separated by a solid Na-ion exchange membrane for grid-scale energy storage. Such HNFBs can operate at ambient temperature, allow catholytes to have multiple electron transfer redox reactions per active ion, offer wide selection of catholyte chemistries with multiple active ions to couple with the highly negative Na alloy anode, and enable the use of both aqueous and non-aqueous catholytes. Further, the molten Na alloy anode permits the decoupled design of power and energy since a large volume of the molten Na alloy can be used with a limited ion-exchange membrane size. In this proof-of-concept study, the feasibility of multi-electron transfer redox reactions per active ion and multiple active ions for catholytes has been demonstrated. The critical barriers to mature this new HNFBs have also been explored.

Energy and climate concerns have led to the development of new renewable energy sources including wind, solar and biofuels. For some of these technologies, such as wind and solar, it is necessary to develop an energy storage system due to the intermittent nature of the power source. Storage is needed so that energy can be stored in times of high production and low demand and released during times of low production and high demand. This is exacerbated by the intermittent nature of some of the sources. Wind turbines have highly variable energy and solar cells have a cyclical energy output. With energy storage it is possible to smooth the output of wind power and extend the usage time of solar. Among various energy storage techniques such as electrochemical storage, compressed gas, pumped hydro and flywheels, electrochemical storage in the form of batteries is the most flexible one^1^. In particular, redox flow batteries (RFBs) are very suitable for grid-scale energy storage owing to their unique advantages including decoupled design of power and energy, no intercalation/deintercalation and stress build-up in electrodes, active heat management due to removal of heat by flowing electrolytes, and capability of storing a large energy/power in a simple design for durations of hours^1^,^2^,^3^,^4^,^5^,^6^,^7^.

The benefits of RFBs mentioned above come from the unique method of energy storage. Li-ion batteries rely on solid state redox reactions, while flow batteries rely on redox reactions taking place in two (2) distinct liquid electrolytes, known as the anolyte and catholyte^2^,^3^,^4^,^5^,^6^,^7^. Different redox chemistries such as S_2_^−2^/S_4_^−2^,^2^,^8^ Cr^2+^/Cr^3+^,^2^,^4^,^9^ and V^3+^/V^2+^,^4^,^6^,^7^,^10^,^11^,^12^,^13^,^14^,^15^,^16^,^17^,^18^,^19^,^20^,^21^,^22^,^23^,^24^ for the anolyte and Fe^2+^/Fe^3+^,^2^,^4^,^25^ Br^3−^/Br^−^,^2^,^5^,^8^,^26^,^27^ VO_2_^+^/VO^2+^,^4^,^7^,^10^,^12^,^15^,^16^,^18^,^19^,^20^,^28^,^29^,^30^,^31^,^32^ Ce^3+^/Ce^4+^,^33^,^34^ and Mn^3+^/Mn^2+^,^2^,^35^,^36^ for the catholyte have been studied. Of the many different RFBs studied, the most common one is the all vanadium RFB. All RFBs suffer from active ion crossover, which limits reversibility as well as cycle life as the active species of the anolyte diffuse to the catholyte and vice versa^10^,^11^,^12^,^14^,^15^,^17^,^19^,^20^,^21^,^23^,^24^,^31^. The all vanadium RFB solves the problem of cycle life associated with ion crossover due to the fact that the vanadium electrolytes can be remixed and charged back to initial condition^2^. The state-of-the-art vanadium RFBs utilize low pH sulfuric acid or mixed acid electrolytes with the vanadium species dissolved inside^37^. Sulfuric acid is used as a source of hydrogen ions for conductivity, and there has been work towards using halides as well to increase stability^4^,^5^,^28^,^37^. Although there has been much progress in improving the vanadium RFB, they are ultimately limited by their energy density. Because the concentration of vanadium species is limited to around 2.5 M and the voltage is limited by the stability window of water to 1.5 V, the vanadium RFBs have low energy density (20–33 Wh/liter) and low specific energy (15–25 Wh/kg)^2^,^28^,^37^,^38^. To improve the energy density of flow batteries, it is necessary to increase the cell voltage or increase the concentration of active ions in the electrolyte^3^,^6^,^13^,^36^,^39^. Organic electrolytes such as acetonitrile have been investigated as higher voltage cells, however they suffer from low concentrations of active species in the organic solvent^6^,^13^. Other approaches include the use of a Li anode separated from the catholyte by a LiSICON membrane^38^ and the semi-solid flow battery in which active solid particles are dispersed in liquid medias that are cycled through the cell^3^. Both approaches can increase the energy density. However, both suffer from different problems. The problem with the Li anode flow batteries is that the power and energy are not truly decoupled because of the solid Li anode. For the semi-solid flow batteries, problems due to the solid electrode are present such as the development of solid electrolyte interphase (SEI) around each active particle, limiting the cycle life. Therefore, there exists the need for a high cycle life and high energy density flow battery that still maintains the separation of power and energy.

In this communication, we introduce a new concept of hybrid Na-based flow batteries (HNFBs) operated at ambient temperature. HNFBs utilize a liquid alkali alloy anode in conjunction with a flowing catholyte to form a high energy, high voltage flow battery (patent pending). As shown in Fig. 1, the battery consist of three (3) major components: the anolyte, the solid electrolyte membrane, and the catholyte. The anolyte is composed of a molten Na alloy or a slurry of solid Na particles dispersed in a molten Na alloy which floats on the Na-ion exchange membrane. The utilization of molten Na alloys allows the specific capacity of the anode to approach the theoretical capacity of Na (1,166 mAh/g). For example, with a Na_90_Cs_10_ alloy the theoretical capacity is 788 mAh/g (assuming no use of Cs for redox reactions). This is about 15 times the specific capacity of a 2.5 M VOSO_4_ (V^4+^) in an aqueous solution (52.7 mAh/g). Furthermore, this Na_90_Cs_10_ alloy is composed of solid Na particles dispersed in a molten Na-Cs alloy at temperature above −8 ^o^C^40^, making it a floating anode at −8 ^o^C or higher. Such a molten anode can be paired with a flowing catholyte to offer a hybrid flow battery with ultrahigh volumetric and gravimetric energy densities while still maintaining the advantage of RFBs in decoupled design of power and energy. The solid ion exchange membrane can be made of β”-Al_2_O_3_ or sodium super ionic conductor (NaSICON) with the ionic conductivity of ~10^−3^ to 10^−5^ S/cm at 25 ^o^C^41^,^42^. The solid membrane allows for the use of both aqueous and non-aqueous catholytes, greatly expanding the options of catholyte chemistries. Because of the use of molten Na alloys at the anode (which has an electrochemical potential of −2.7 V vs SHE), the cell voltage can be increased to 3 volts or higher, depending on the catholyte chemistry. This further increases the energy density of HNFBs by a factor of 3 in comparison with conventional RFBs using an aqueous media for both the anolyte and catholyte. The aqueous solutions have limited the operating voltage of conventional RFBs to around 1.3 volts^2^,^28^,^37^,^38^.

The catholyte chemistry can be the same as or similar to that of traditional RFBs. However, due to the highly negative potential of the molten anode, more redox chemistries of the catholyte can be explored. In particular, redox systems in traditional RFBs that are used as the anolyte can now be used as the catholyte. For example, the reactions in both the anolyte and catholyte in all vanadium RFBs can potentially be used in the catholyte of HNFBs, as shown by reactions (1) to (3) below.
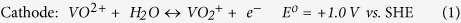

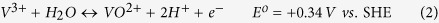

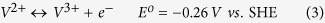

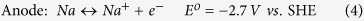
Because all the vanadium reactions can be used as a catholyte, it is possible to cycle a vanadium based catholyte between V^2+^ and V^5+^, with each transition providing one electron for a total of 3 electron transfers per V ion. Based on these redox reactions of the catholyte and assuming that the catholyte contains 2.5 M active V ions, one can obtain a theoretical specific energy of 483.7 Wh/kg, which is the sum of redox 1 (195 Wh/kg), redox 2 (160 Wh/kg), and redox 3 (128.7 Wh/kg) reactions. This specific energy (480 Wh/kg) is 18 times the specific energy provided by conventional all vanadium RFBs (~25 Wh/kg)^2^.

In addition to multiple electron transfer redox reactions per active ion, HNFBs also allow catholytes with multiple active ions to increase the energy density. The concept of multiple active ions has been demonstrated before^43^, but with the much lower electrochemical potential of the anode in HNFBs there are more options to select effective and compatible multiple active ions to increase the energy density. In what follows, the results from the proof-of-concept studies for multi-electron transfer redox reactions per active ion are presented.

## Results

### HNFBs with Non-Aqueous Catholytes

**Figure 2:**
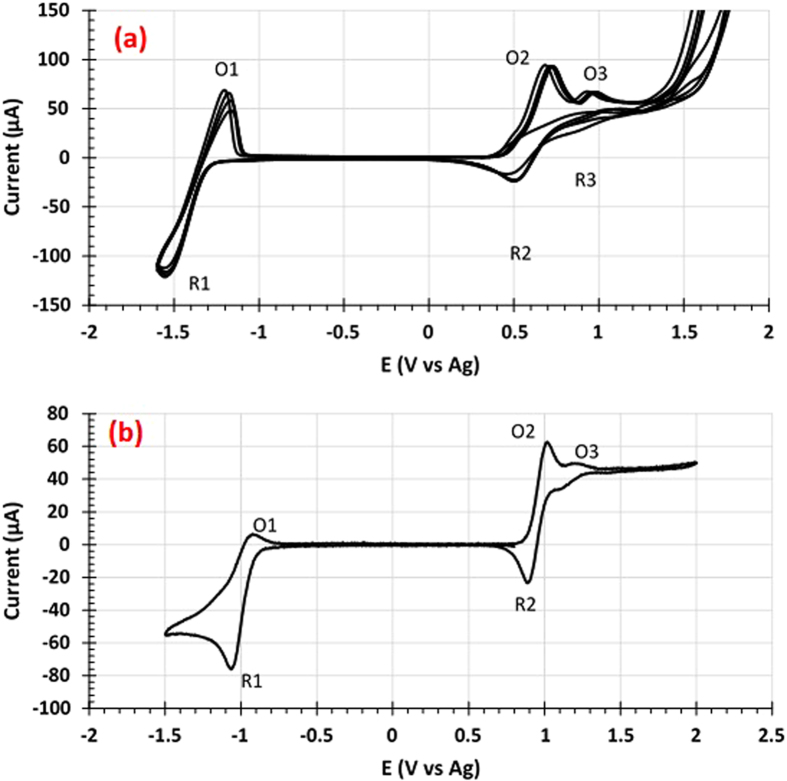
**CV curves** (**a**) for 0.025 M V(acac)_3_ with 0.1 M NaClO_4_ and (**b**) for 0.01 M V(acac)_3_ with 0.05 M NaPF_6_ in acetonitrile, measured on a glassy carbon electrode with a scan rate of 10 mV/s.

Vanadium acetylacetonate, V(acac)_3_, dissolved in acetonitrile (CH_3_CN) with supporting electrolytes of 0.1 M NaClO_4_ and 0.05 M NaPF_6_ is studied for its potential to offer more than one electron transfer redox reaction per V ion. The concentration of V(acac)_3_ in these solutions are 0.025 M and 0.01 M for the catholytes with 0.1 M NaClO_4_ and 0.05 M NaPF_6_ supporting electrolytes, respectively. The cyclic voltammogram (CV) of these two types of catholytes are shown in Fig. 2. Note that the two catholytes display similar CV profiles, showing three oxidation peaks (O1, O2 and O3 marked in both CV profiles) and three reduction peaks (marked as R1, R2 and R3). These CV profiles are also similar to the CV profile measured previously using the solution with 0.01M V(acac)_3_ in acetonitrile with 0.5 M TEABF_4_ supporting electrolyte^6^. O1 and R1 have been identified to represent the V^2+^/V^3+^ transition, whereas O2 and R2 to represent the V^3+^/V^4+^ transition^6^. O3 and R3 have not been unambiguously assigned yet but could stem from the V^4+^/V^5+^ transition or the oxidation of the V(acac)_3_ species to VO(acac)_2_^6^. The weak R3 peak displayed by both catholytes (Fig. 2a,b) suggests that the redox reactions of O3 and R3 are not very reversible. Finally, it is noted that the supporting electrolyte has some effect, although small, on the peak position and peak current. It appears that the NaClO_4_ supporting electrolyte has moved all of the redox peaks to lower electrochemical potentials with respect to the catholyte containing NaPF_6_.

**Figure 3:**
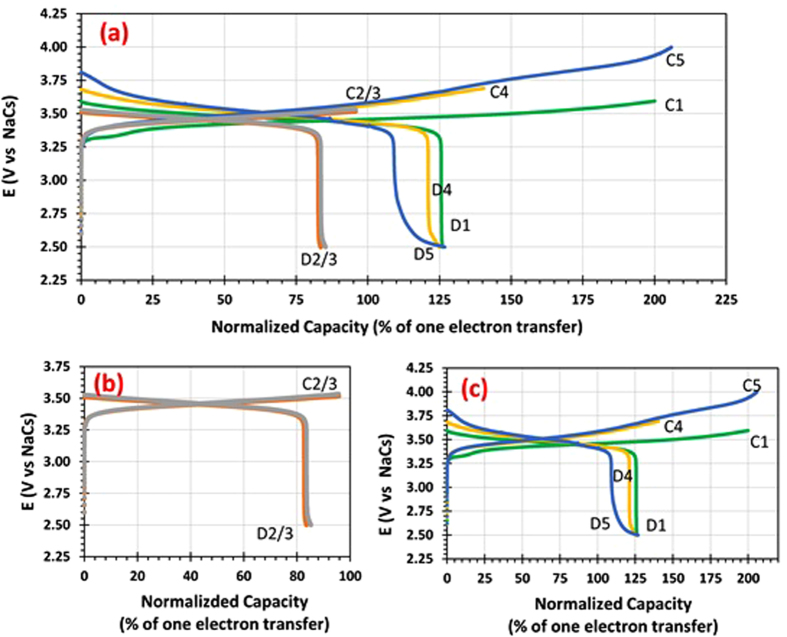
**Performance of the catholyte made of 0.005 M V(acac)_3_ with 0.05 M NaPF_6_ in acetonitrile coupled with a NaCs anode charged to various capacities and discharge to 2.5 V at a current of 0.05 mA, showing** (**a**) all 5 cycles, (**b**) a closer look of cycles 2 and 3, and (**c**) a closer look of cycles 1, 4 and 5.

The galvanostatic charge-discharge curves of HNFBs with a molten Na_0.37_Cs_0.63_ alloy placed inside a tubular β”-Al_2_O_3_ membrane and the catholyte made of 0.005M V(acac)_3_ with 0.05M NaPF_6_ in acetonitrile and placed outside the β”-Al_2_O_3_ tube are shown in Fig. 3. To focus on studies of more than one electron transfer redox reactions per active ion, the x-axes for all of the charge-discharge curves in this communication are plotted based on the percentage of the theoretical value for one electron transfer per V ion. Further, the concentration of V(acac)_3_ in the catholyte is kept low to reduce the testing time. The volume of the catholyte is minimized too in order to get experiments with multiple charge/discharge cycles completed in less than one week rather than months. To accomplish the goal of completing experiments in less than one week, we have designed a special setup that can allow us to stir the catholyte (see Supplementary Materials, Figure S1). This setup permits us to maintain the feature of a “flowing catholyte” while reducing the volume of the catholyte to a minimum.

There are several distinct features that can be seen in Fig. 3. The first operation for this cell is charging, and thus the first redox reaction for the catholyte is from V^3+^ to V^4+^ transition since the catholyte starts at the V^3+^ state. The first charge continues until the charge capacity of the catholyte has reached 2 electron transfer redox reactions (with the cell voltage changes from 3.32 V to 3.62 V). It is noted that there is no voltage jump at the transition from one electron transfer redox reaction (corresponding to V^3+^ to V^4+^) to the second electron transfer redox reaction (corresponding to V^4+^ to V^5+^ or V(acac)_3_ to VO(acac)_2_). The lack of the voltage jump is not a surprise since the CV data (Fig. 2b) shows significant overlap between O2 and O3 peaks. Upon the first discharge, the cell voltage changes from 3.60 V to 2.50 V. The very small voltage change (−0.02 V) upon switching the current direction indicates a very small overpotential for discharge. However, the Coulombic efficiency is only 63.0%. Thus, for the second and third charge operation, charging is only carried out within the one electron transfer redox reaction range (i.e., 96% of the theoretical value for one electron transfer per V ion). The Coulombic efficiency improves to 87.3% and 89.1% for the second and third charge/discharge cycles, respectively (Fig. 3b). Further, the second and third charge/discharge curves almost overlap. All of these indicate that the V^3+^/V^4+^ transition is very reversible, consistent with the CV data (Fig. 2b).

To find out whether the low Coulombic efficiency of the first cycle is due to the lower reversibility of O3/R3 redox reactions (Fig. 2b) or the “activation” of the cell often observed for many Li-ion batteries, the 4^th^ and 5^th^ charges are conducted beyond the one electron transfer limit again with the 4^th^ charge reaching 135% of the theoretic value for one electron transfer per V ion and the 5^th^ charge reaching 210% of the theoretic value for one electron transfer per V ion (Fig. 3c). It is interesting to note that the Coloumbic efficiency of the 4^th^ cycle is 89.0%, whereas the corresponding value for the 5^th^ cycle is only 61.2%. These data reveal that the Coloumbic efficiency is low if charge is beyond 135% of the theoretic value for one electron transfer per V ion. This finding is in good accordance with the CV measurement (Fig. 2b) which exhibits low reversibility of O3/R3 reactions.

**Figure 4:**
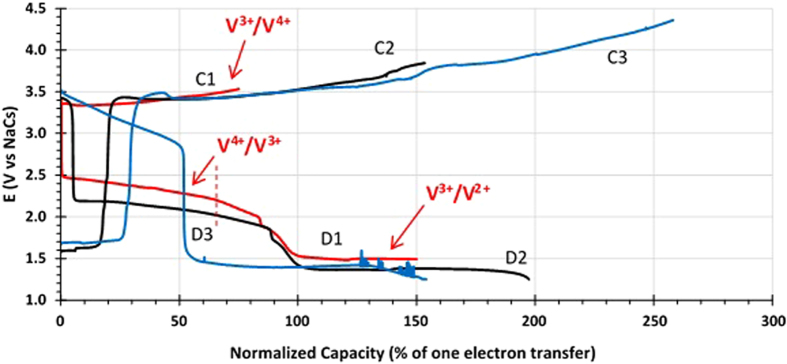
Charge/discharge performance of the catholyte made of 0.005 M V(acac)_3_ with 0.05 M NaPF_6_ in acetonitrile coupled with a NaCs anode: Cycle 1 (red) charged 75% from V^3+^ to V^4+^ and discharged 150% from V^4+^ to V^3+^ and then V^3+^ to V^2+^ at 0.1 mA; Cycle 2 (black) charged 153% from V^2+^ to V^3+^ and then V^3+^ to V^4+^ and discharged 197% from V^4+^ to V^3+^ and then V^3+^ to V^2+^ at 0.25 mA; and Cycle 3 (Blue) charged 248% from V^2+^ to V^3+^ and then V^3+^ to V^4+^ and discharged 153% from V^4+^ to V^3+^ and then V^3+^ to V^2+^ at 0.25 mA. The redox couple for each plateau of Cycle 1 is indicated, whereas other cycles are not marked for clarity. See the text for discussion.

The feasibility of utilizing V^2+^/V^3+^ (O1/R1) and V^3+^/V^4+^ (O2/R2) redox reactions (Fig. 2b) to offer more than one electron transfer redox reactions per V ion for V(acac)_3_ with 0.05 M NaPF_6_ in acetonitrile has also been investigated using the same setup as shown in Figure S1. In this set of experiments, the cell is charged first from V^3+^ (the starting state) to V^4+^ up to 75% of the theoretical value of one electron transfer (Fig. 4). Then the cell is discharged 150% from V^4+^ to V^3+^ and then V^3+^ to V^2+^. Upon switching the current direction from charge to discharge, we notice that the cell voltage decreases from 3.55 V to 2.50 V right away, suggesting overpotential in discharge or the cell still in the “activation” stage. At about 65% discharge marked by the vertical dash line, the slope of the discharge curve changes, indicating the transition from the V^3+^/V^4+^ couple to the V^2+^/V^3+^ couple reaction. The continued discharge finally ends up at a cell voltage of about 1.5 V. Thus, the voltage difference between the V^3+^/V^4+^ and V^2+^/V^3+^ redox couples of the full cell is about 2 V, in excellent agreement with the CV data shown in Fig. 2(b).

The second charge starts at the cell voltage of ~1.6 V, a small increase from the discharge voltage of 1.5 V and thus a low overpotential for the V^2+^ to V^3+^ oxidation. However, the V^2+^/V^3+^ redox reaction only last about 20% at which the cell voltage rises to the voltage of ~3.4 V corresponding to the V^3+^/V^4+^ redox reaction voltage. This result indicates that the V^2+^/V^3+^ redox reaction is not very reversible. Such a low reversibility of the V^2+^/V^3+^ redox reaction persists in the third charge cycle. In the third charge operation, the cell has been charged to beyond the theoretical value of two electron transfer (260%). Upon switching the current direction from charge to discharge, the cell voltage drops from ~4.4 V to 3.5 V immediately. Further, the discharge capacity corresponding to the combined V^5+^/V^4+^ and V^4+^/V^3+^ redox reactions is very low (only 50% of one electron transfer), while the charge capacity is about 225%. Thus, the Coulombic effecience is only 22%, most likely due to significant side reactions at the cell voltage higher than 4 V and the poor reversibility of the V^5+^/V^4+^ redox reaction as revealed in the previous experiment (Fig. 3).

It should be mentioned that in charge/discharge experiments, the color of the catholyte varies in response to the cell voltage (Figure S2), indicating that the valence state of V ions has been altered with the cell voltage. Changing color of V ions with its oxidation state is a well-known phenomenon^44^. Thus, this phenomenon serves as an effective visual aid in judging whether the valence state of V ions has altered or not as the cell voltage changes.

Both sets of the experiments (Figs 3 and 4) unambiguously demonstrate that the new concept of HNFBs can allow the utilization of multi-electron transfer redox reactions per V ion to increase the energy density of hybrid Na-based flow batteries. However, the Coulombic efficiencies and cycle stability for V^2+^/V^3+^ and V^5+^/V^4+^ redox reactions need to improve significantly. In contrast, the V^3+^/V^4+^ redox reaction appears to have a high Coulombic efficiency and good cycle stability (Fig. 3b).

### HNFBs with Aqueous Catholytes

**Figure 5:**
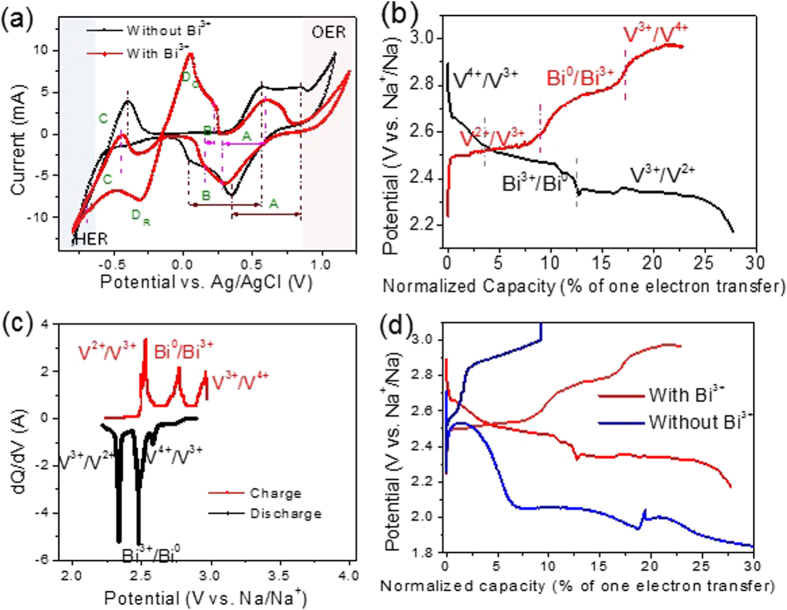
(**a**) CV curves of aqueous electrolytes with and without BiCl_3_ in half cells at a scan rate of 10 mV/s in which a graphite felt, Ag/AgCl, and Pt wire were used as the working, reference, and counter electrodes, respectively; (**b**) the charge/discharge profile (1^st^ cycle) of a full cell at 0.02 mA with the 0.01 M VOSO_4_ −0.05 M Na_2_SO_4_ −1.5 M HCl −0.002 M BiCl_3_ aqueous solution as the catholyte and the Na chip anode with the 0.5 M NaTFSI / pyrrTFSi electrolyte in which the three charge/discharge plateaus are identified; (**c**) the differential capacity vs. voltage of the charge/discharge profile in (**b**); and (**d**) the comparison of the charge/discharge profiles between the full cells with and without BiCl_3_ in catholytes.

The feasibility of using the aqueous catholyte in HNFBs is evaluated using VOSO_4_ (V^4+^) dissolved in a acid solution (with Na_2_SO_4_ + HCl). Theoretically, as indicated by reactions (1) to (3), VO^2+^(IV) can be reduced to V^3+^ (III) and then V^2+^(II) or oxidized to VO_2_^+^(V), giving redox potentials of ~3.04, 2.44, 3.70 V vs. Na^+^/Na, respectively. Fig. 5(a) shows the stable electrochemical potential window for the acidic aqueous catholyte solution (0.01M VOSO_4_ and pH ~1) and the redox potential of V ions. Li, *et al*^45^. have found that the addition of BiCl_3_ in acidic V aqueous electrolytes could improve the reversibility of V ion redox reactions. Thus, we have compared the CV curves of the catholytes with and without BiCl_3_. The A, B, C redox peaks are attributed to the V^4+^/V^5+^, V^3+^/V^4+^, and V^2+^/V^3+^ redox reaction couples, respectively, while the strong D peaks are generated by the redox reaction of Bi^3+^/Bi^0^. However, the peak of V^3+^/V^2+^ reduction (C_R_) are smeared due to the water electrolysis. It can be clearly seen that after adding BiCl_3_, the peak potential separations corresponding to redox reactions V^3+^/V^4+^ and V^4+^/V^5+^ are decreased by 420 and 200 mV, respectively, suggesting that Bi^3+^ have electrocatalytic effect on redox reactions. Although the previous study^45^ reveals that Bi^0^ in the Bi(III)/Bi(0) couple has electrocatalytic effect only on V^2+^/V^3+^ redox reaction, in our case the electrocatalytic effect of Bi^3+^ on V^3+^/V^4+^ and V^4+^/V^5+^ are more significant.

Figure 5(a) also shows that the O_2_ evolution reaction (OER) will not occur on carbon foam when potential < 0.9 V vs. Ag/AgCl. However, the reduction of V^3+^ to V^2+^ is slightly overlap with the H_2_ evolution reaction (HER) when the potential is more negative than −0.6 V vs. Ag/AgCl. Thus, the charge/discharge investigation of HNFBs with the VOSO_4_-based aqueous catholyte can be carried out in the potential window from −0.55 V to 0.9 V vs. Ag/AgCl (i.e., 2.35–3.8 V vs Na^+^/Na). Considering the overpotential in the cell, the practical threshold can be expanded to 2.2–4.0 V vs. Na^+^/Na, which is the potential window for most tests in the aqueous catholyte cases. We believe that water electrolysis could not take place within this potential range.

The charge/discharge behavior of the HNFB with the aforementioned aqueous catholyte and a flat β”-Al_2_O_3_ membrane are examined and shown in Fig. 5(b). The current density was set to 5 μA/cm^2^ in consideration of the low conductivity at room temperature (~7 × 10^−5^ S/cm, see Supplementary Materials, Figure S6) and the thickness (1.4 mm) of the solid electrolyte. The setup of the cell for evaluating the aqueous catholyte can be found in Figure S3. Similar to the evaluation of non-aqueous catholytes, the catholyte is stirred to permit the use of a small quantity of the catholyte so that the charge/discharge experiment can be completed in less than one week. Three plateaus can be observed on the 1^st^ cycle of both discharge and charge curves, corresponding to three different redox reactions. The test starts from discharge. Based on the starting valence state of the V ion (IV) and the CV curves of the corresponding catholyte (Fig. 5a), these plateaus can be identified to correspond to the redox reactions of V^4+^(IV)/V^3+^(III), Bi^3+^(III)/Bi(0), and V^3+^(III)/V^2+^(II) as labelled in the figure. Therefore, for the first time the concept of multiple electron transfer redox reactions per active ion in aqueous electrolytes (two electron transfer per V active ion in this case) has been validated. Moreover, the result shows multiple electron transfer obtained by two active species, i.e. V and Bi ions. Here, the V ion plays as the main active ion, while Bi acts both as an active ion and a catalyst.

To further confirm the nature of the three plateaus in Fig. 5(b), the corresponding dQ/dV (differential capacity) vs. voltage curves are examined and plotted in Fig. 5(c). The differential capacity peaks match the CV curve very well, giving 2.45 V for the V^2+^/V^3+^ redox reaction and 2.95 V for the V^3+^/V^4+^ redox reaction. Moreover, it shows the peak potential separation between V^2+^/V^3+^ and V^3+^/V^4+^ being ~0.6 V and the peak potential separation between V^2+^/V^3+^ and Bi^3+^(III)/Bi(0) being ~0.4 V, directly confirming the identification of these plateaus. The differential capacity peaks in the charge curve are much closer to the theoretical values (with only ± 0.1 V deviation) than those in the discharge curve (−0.2 to −0.4 V deviation), suggesting larger overpotentials in the discharge process.

The electrochemical catalytic effect of adding BiCl_3_ can be seen clearly from Fig. 5(d). The battery with the BiCl_3_-containing catholyte exhibits a much smaller potential gap between the charge and discharge plateaus than the battery without BiCl_3_, unambiguously indicating the catalytic effect of BiCl_3_ in reducing overpotentials for redox reactions. This phenomenon is consistent with the CV data (Fig. 5a).

It should be pointed out the low capacity (10–20% vs. theoretical one electron transfer capacity) for each redox reaction shown in Fig. 5(b,d) is mainly due to the insufficient mass transportation in the catholyte. As shown in Figure S3, the catholyte was only stirred at the lower portion of the catholyte chamber using a 3-mm (length) mirco-stir bar. Moreover, the thick (~5 mm), porous graphite felt electrode make the mass transportation inside the felt slow and difficult. Thus, the lower portion catholyte has not been fully utilized for redox reactions, leading to early transition from one redox couple to another as shown in Fig. 5(b,d). In spite of this limitation, the present experimental setup permits us to work with a small quantity of the catholyte and complete the experiment in less than one week. If the setup of a flowing catholyte had been used, one experiment could have taken months to complete.

**Figure 6:**
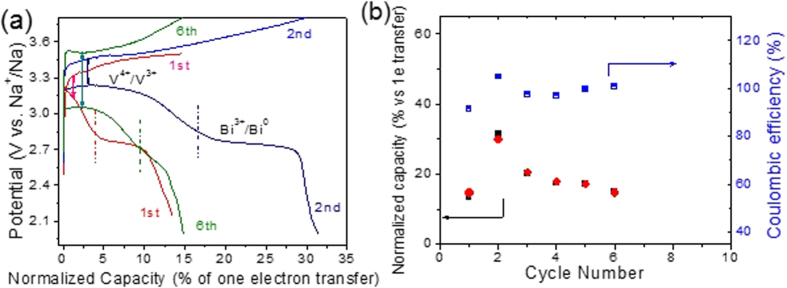
Charge/discharge capacity versus theoretical capacity of one electron transfer per V ion and the corresponding Coulombic efficiency at 0.02 mA at room temperature over cycling of a stationary battery. Black solid squares and red solid spheres refer to the discharge and charge normalized capacities, respectively.

The cyclability and stability of the aqueous catholyte have been further examined in batteries without stirring of the catholyte. Their representative capacities and Coulombic efficiency for each step up to 6 cycles have been illustrated in Fig. 6. The experiment is initiated with the discharge test. The charge/discharge profile shows the redox reaction plateaus for V^4+^/V^3+^ and Bi(III)/Bi(0), separated and marked by the dotted line. Charge/discharge capacity increase at the second cycle is due to the improved interface contacts of the catholyte with the graphite felt electrode^38^,^46^ and with the β”-Al_2_O_3_ membrane, both of which have reduced the overpotential of the cell in the 2^nd^ cycle. However, beyond the 2^nd^ cycle, the capacity gradually decays over cycles which could be ascribed to increased overpotential with cycling. The potential discrepancy between the charge and discharge plateaus for the V^4+^/V^3+^ redox reactions rises from ~0.15 V to 0.45 V when the cycle number increases from the 1^st^ to 6^th^ cycle. Upon further looking into the charge/discharge profile, the major capacity fading is at the Bi(III)/Bi(0) plateau, indicating the loss of the Bi nanoparticles on the carbon electrode and/or aggregation of the Bi nanoparticles. The loss on the concentration and fine distribution of Bi on the carbon electrode have been previously confirmed responsible for the loss of catalytic activity towards V redox reactions^45^. The Coulombic efficiency are ~100% after the 2^nd^ cycle, suggesting that the gradual decay in the capacity is likely related to the decay of the catalytic activities because of the loss of the Bi nanoparticles on the carbon electrode and/or aggregation of the Bi nanoparticles.

## Discussion

In this proof-of-concept study, we have introduced a new concept of hybrid Na-based flow batteries (HNFBs) with a floating liquid Na alloy anode in conjunction with a flowing catholyte separated by a solid Na-ion exchange membrane. We have demonstrated that such a hybrid Na-based flow battery can operate at ambient temperature, allow catholytes to have multiple electron transfer redox reactions per active ion, offer wide selection of catholyte chemistries with multiple active ions to couple with the highly negative Na alloy anode, and enable the use of both aqueous and non-aqueous catholytes. Further, the molten Na alloy anode permits the decoupled design of power and energy since a large volume of the molten Na alloy can be used with a limited ion-exchange membrane size. Owing to the combined effectiveness of these unparalleled features, HNFBs can have significantly higher energy densities than conventional RFBs while still maintaining the advantages of conventional RFBs, including separation of power and energy, operation at ambient temperature, active heat management due to removal of heat by flowing electrolytes, no intercalation/deintercalation and stress build-up in electrodes, and capability of storing a large energy/power in a simple design for durations of hours.

This proof-of-concept study has also identified several critical issues that need to be overcome in order to make HNFBs a viable technology. The two most critical issues identified are (a) the cycling stability and low Coulombic efficiency of catholytes with multiple electron transfer redox reactions per active ion and (b) the low ionic conductivity and mechanical strength of the solid Na-ion exchange membranes. All of the catholytes, both aqueous and non-aqueous, investigated in this study exhibit the problem of cycling stability and low Coulombic efficiency when more than one electron transfer redox reactions per active ion are explored. One of the effective approaches to address these issues is to study effective catalysts. As shown in Figure S4, addition of Bi^3+^ ions to the catholyte can significantly improve the reversibility of multiple V ion redox reactions. Another important way to enhance the cycling stability and Coulombic efficiency is to control the surface chemistry or functionalize the surface of the electrode. By comparing the CV curves of Fig. 5(a) and S4, it can be concluded that the graphite felt is much more effective serving as the electrode to promote V ion redox reactions than the glassy carbon. We attribute this phenomenon to difference in the surface conditions between the graphite felt and glassy carbon because both are good electronic conductors.

The low ionic conductivity and mechanical strength of the existing solid Na-ion exchange membranes could also impose limitations on the ultimate performance of HNFBs. This issue, however, is less acute for the moisture-sensitive β”-Al_2_O_3_ membranes since they have relatively high Na ion conductivity (2 × 10^−3^ S cm^−1^ at 25 ^o^C)^41^ and thickness of the β”-Al_2_O_3_ tube is 0.5 mm, making the resistance of the membrane low. Indeed, our electrochemical impedance spectroscopy (EIS) measurements reveal that the IR resistance of the full cell shown in Figure S1 is low. As shown in Figure S5, the electronic resistance R_e_ (~35 Ω) and charge transfer resistance R_ct_ (~54 Ω) derived from the equivalent circuit of the full cell are both quite small. The linear tail at low frequency is mainly due to the cathode acting as a double layer capacitor. It acts as a double layer capacitor because the initial OCV is between the V^3+/4+^ and V^2+/3+^ potentials.

In sharp contrast, the moisture-resistant β”-Al_2_O_3_ membranes have low Na ion conductivity (7 × 10^−5^ S cm^−1^ at 25 ^o^C, see Figure S6) which is two orders of magnitude lower than the ionic conductivity of most of the conventional liquid electrolytes. Furthermore, the commercially available moisture-resistant β”-Al_2_O_3_ membranes have large thickness (~1.4 mm). Therefore, one would expect the full cell built with moisture-resistant β”-Al_2_O_3_ membranes to have high resistance. Thus, improvements in enhancing the ionic conductivity and reducing the thickness of the moisture-resistant β”-Al_2_O_3_ membranes are necessary in the future to enable HNFBs to operate with aqueous catholytes.

The final aspect that needs to be considered is the stability of β”-Al_2_O_3_ membranes with water. It is well known that β”-Al_2_O_3_ is prone to moisture due to the presence of a small amount of hygroscopic sodium aluminate (NaAlO_2_) at grain boundaries^48^. Using a vapor phase conversion method, this NaAlO_2_ phase can be eliminated from grain boundaries, leading to the moisture-resistant β”-Al_2_O_3_ membranes^48^. In this study, we have conducted X-ray diffraction (XRD) measurements of membranes before and after charge/discharge cycles for 30 days in acidic electrolytes. As shown in Figure S7, no discernible changes are present. However, the long-term stability of these β”-Al_2_O_3_ membranes remains to be tested.

## Methods

### Assembly of HNFBs

All chemicals were purchased from Sigma Aldrich, except BiCl_3_ and H_2_SO_4_ which were from from Alfa Aesar. They were all used as received. Sodium bis(trifluoromethylsulfonyl)imide (NaTFSI) was synthesized via bis(trifluoromethane)sulfonimide (TFSI) as reported^47^. The cell structures are shown in Figures S1 and S3 for HNFBs with the non-aqueous and aqueous catholytes, respectively. The anode was made of (a) a liquid Na-Cs alloy as the electrode and a metal wire with a Na chunk as the current collector (Figure S1), or (b) a metal wire with a Na chunk as the electrode and current collector submerged in a 0.1~1.0 M solution of NaTFSI in 1-Butyl-1-methylpyrrolidinium bis(trifluoromethylsulfonyl)imide (pyrrTFSi) (Figure S3). The liquid Na-Cs alloy had a formula of Na_37_Cs_63_ prepared by mixing two metals, heating up above their melting points, and cooling down to room temperature.

On the cathode side, either an aqueous catholyte or a non-aqueous catholyte was used, and a graphite felt was uesd as the current collector. For non-aqueous catholytes, two systems were studied with one being 0.025 M V(acac)_3_ (V^3+^) electrolytes with 0.1 M NaClO_4_ as the supporting electrolyte dissolved in acetonitrile, and the other being 0.01 M V(acac)_3_ electrolytes with 0.05 M NaPF_6_ as the supporting electrolyte also dissolved in acetonitrile. For aqueous catholytes, VOSO_4_ (V^4+^) with Na_2_SO_4_ and HCl in an aqueous solution with varied concentrations were investigated.

The anode and cathode were separated by a β”-Al_2_O_3_ solid electrolyte. Water-resistant β”-Al_2_O_3_ discs, prepared via a vapor phase synthesis^48^ (Materials and Systems Research, Inc., 1.4 mm thick with an ionic conductivity of 7 × 10^−5^ S cm^−1^ at 25 °C (Figure S6) was used for HNFBs with aqueous catholytes, while moisture-sensitive β”-Al_2_O_3_ tubes (Ionotec Ltd., 0.5 mm thick with an ionic conductivity of 2 × 10^−3^ S cm^−1^ at 25 °C)^41^ was employed for HNFBs with non-aqueous catholytes. The effective area of the solid electrolyte was ~3.8 cm^2^ for HNFBs with aqueous catholytes, and 4.6 to 5.7 cm^2^ in HNFBs with non-aqueous catholytes, depending on the immersion depth of the β”-Al_2_O_3_ tube into the catholyte liquid.

The cell assembling was carried out in an argon-filled glovebox with moisture level lower than 0.1 ppm and oxygen level lower than 1 ppm. The only exception to this procedure was the aqueous catholyte which was incorporated to HNFBs in air.

### Electrochemical analysis and characterization

The HNFBs with aqueous catholytes were activated by flowing the catholyte for 1 hour followed by charge/discharge for 12 h before the formal charge/discharge operation to ensure full soakage of the solution into every part. For the HNFBs with non-aqueous catholytes, a current of 0.01 –0.2 mA was applied to galvanostatically cycle the battery between 1.5 V and 4.8 V (using Parstat 4000, Princeton Applied Research or Neware battery testing system), while for the HNFBs with aqueous catholytes the current applied was 0.02 mA.

Three electrode setups were used for the multi-scan cyclic voltammetry (CV) test using Parstat 4000. In the CV test with non-aqueous catholytes, a glassy carbon (Gamry Instruments), Pt wire, and Ag wire in a glass pipet served as the working, counter and quasi reference electrode, respectively. For the CV test of aqueous electrolytes, a graphite felt (or a glassy carbon) and Pt wire were used as the working and counter electrodes along with an Ag/AgCl reference electrode.

Potentiostatic EIS measurements were conducted at the open circuit voltage (OCV). A sinusoidal signal with amplitude of 10 mV was applied to the battery. The frequency of the signal scanned ranged from 100 kHz to 1 Hz for aqueous cells and 10 kHz to 0.1 Hz for non-aqueous cells. All tests were performed at room temperature with Parstat 4000.

## Additional Information

**How to cite this article**: Shamie, J. S. *et al.* Room Temperature, Hybrid Sodium-Based Flow Batteries with Multi-Electron Transfer Redox Reactions. *Sci. Rep.* **5**, 11215; doi: 10.1038/srep11215 (2015).

## Supplementary Material

Supplementary Information

## Figures and Tables

**Figure 1:**
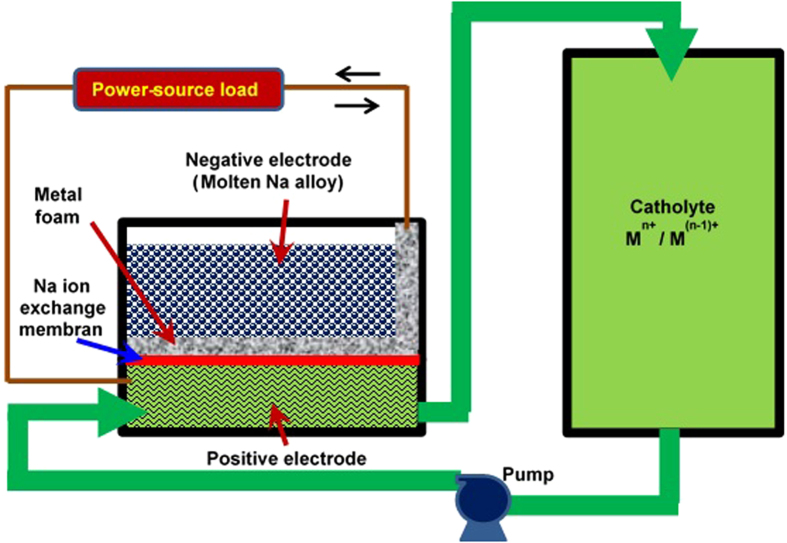
Schematic of hybrid Na-based flow batteries (HNFBs) with a floating anode on the Na-ion exchange membrane and a flowing catholyte, operated at ambient temperature.
