# Supplementary material for: Room Temperature, Hybrid Sodium-Based Flow Batteries with Multi-Electron Transfer Redox Reactions
Source: Sci Rep. 2015 Jun 11;5:11215. doi: 10.1038/srep11215 (PMC4463013; doi:10.1038/srep11215)
Supplement: Supplementary Information [file srep11215-s1.pdf]

Supplementary Information

Room Temperature, Hybrid Sodium-Based Flow Batteries with Multi-Electron Transfer Redox Reactions

Jack S. Shamie,[a,b] **Caihong Liu,**[a,b] **Leon L. Shaw,** [a,b,1] **Vincent L. Sprenkle** [c]

[a] Wanger Institute for Sustainable Energy Research

[b] Department of Mechanical, Materials and Aerospace Engineering

Illinois Institute of Technology, Chicago, Illinois 60616

[c] Energy Storage and Conversion Energy Materials

Pacific Northwest National Laboratory, Richland, WA 99352

[1] Corresponding author: Leon Shaw, lshaw2@iit.edu

S1: Cell setup for evaluation of non-aqueous catholytes

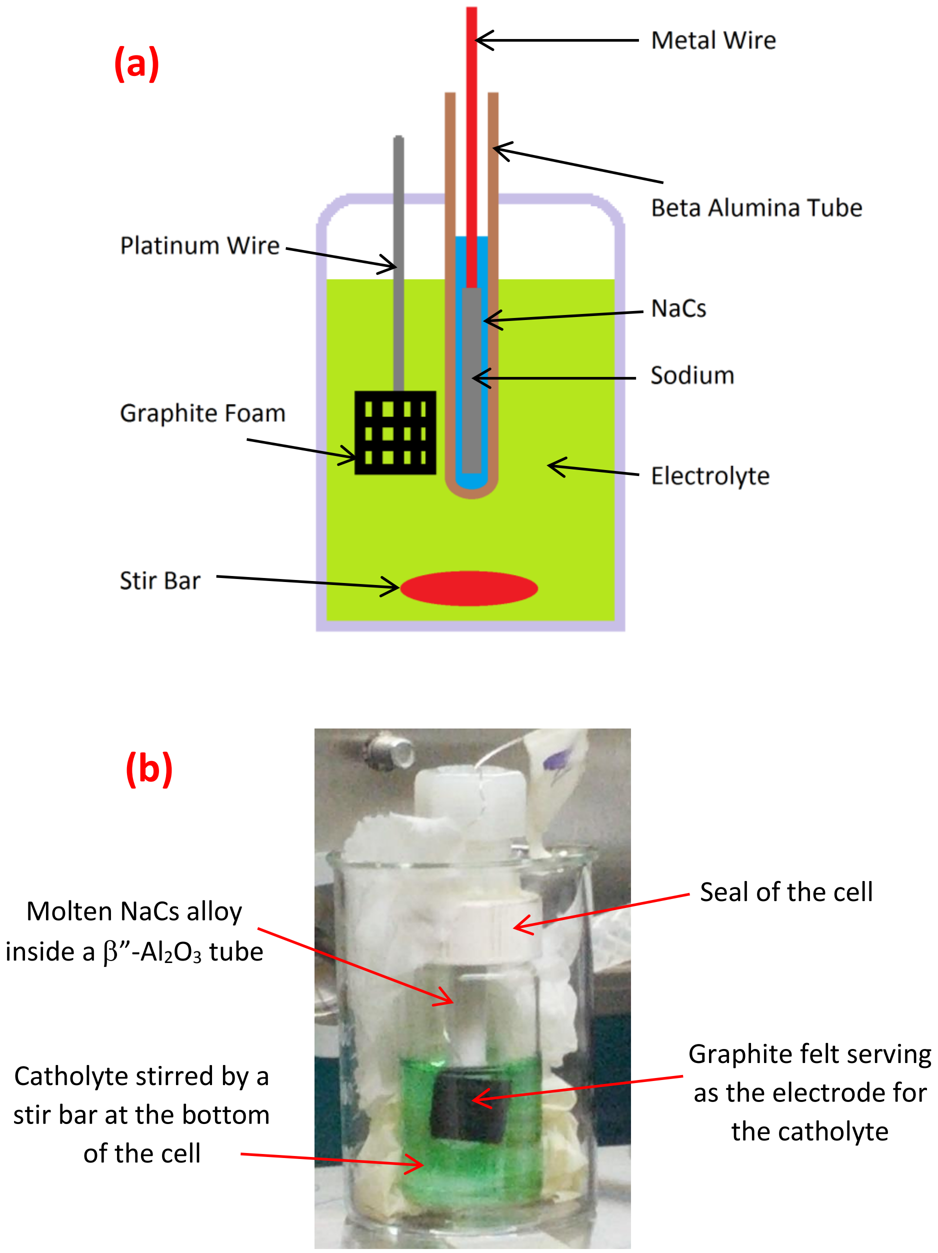

Figure S1: (a) Schematic of the cell setup for evaluating non-aqueous catholytes with more than one electron transfer redox reactions, and (b) a photo of the real cell setup. The dimensions of the cell are about 120 mm height and 75 mm in diameter.

S2: Color change of the catholyte as the cell potential changes

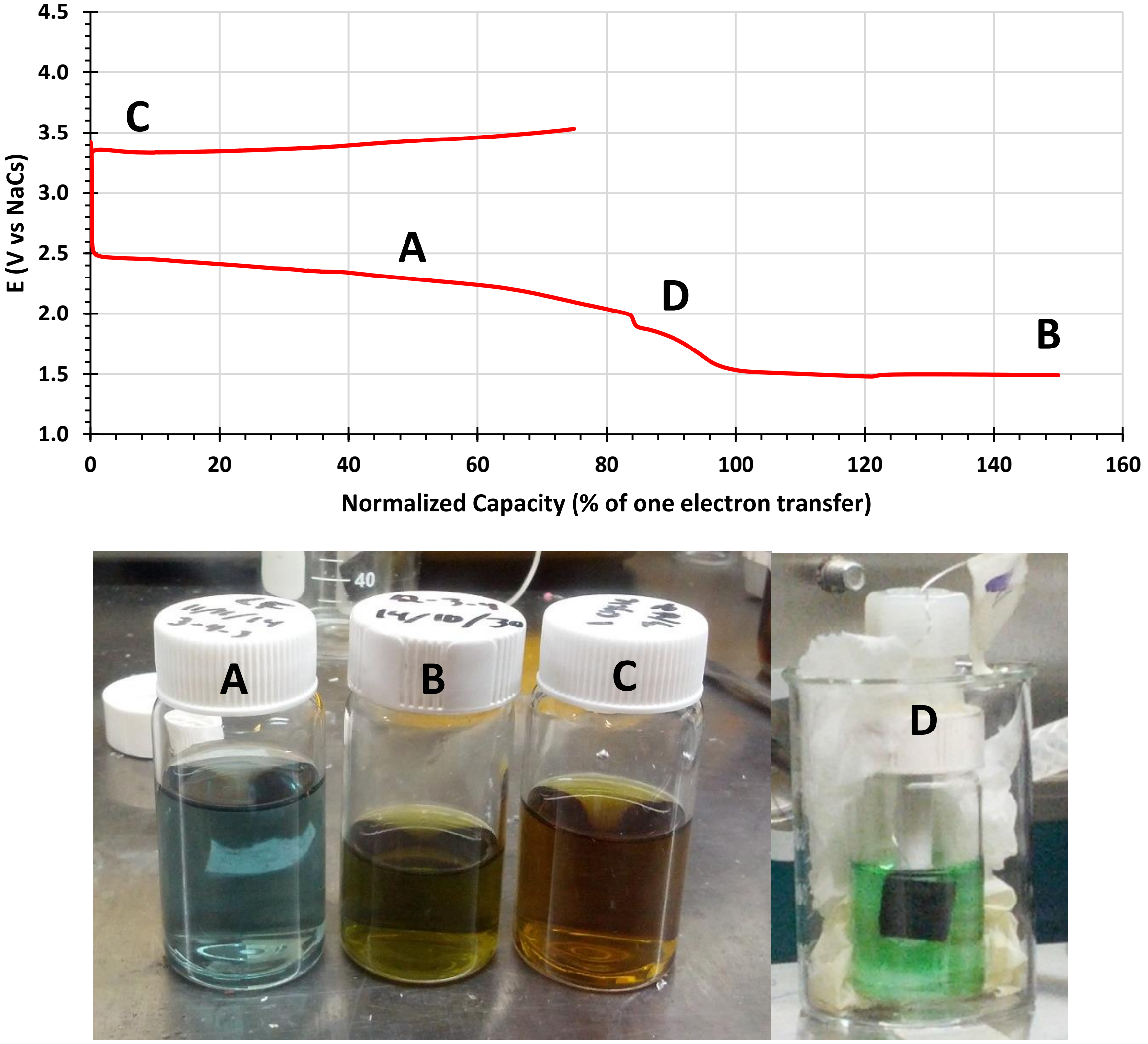

Figure S2: Color change of the catholyte as the cell potential changes. The catholyte is made of 0.005M $V(acac)_3$ with 0.05M $NaPF_6$ in acetonitrile, and coupled with a NaCs anode. The corresponding state of each color is marked in the charge/discharge curves.

S3: Cell setup for evaluation of aqueous catholytes

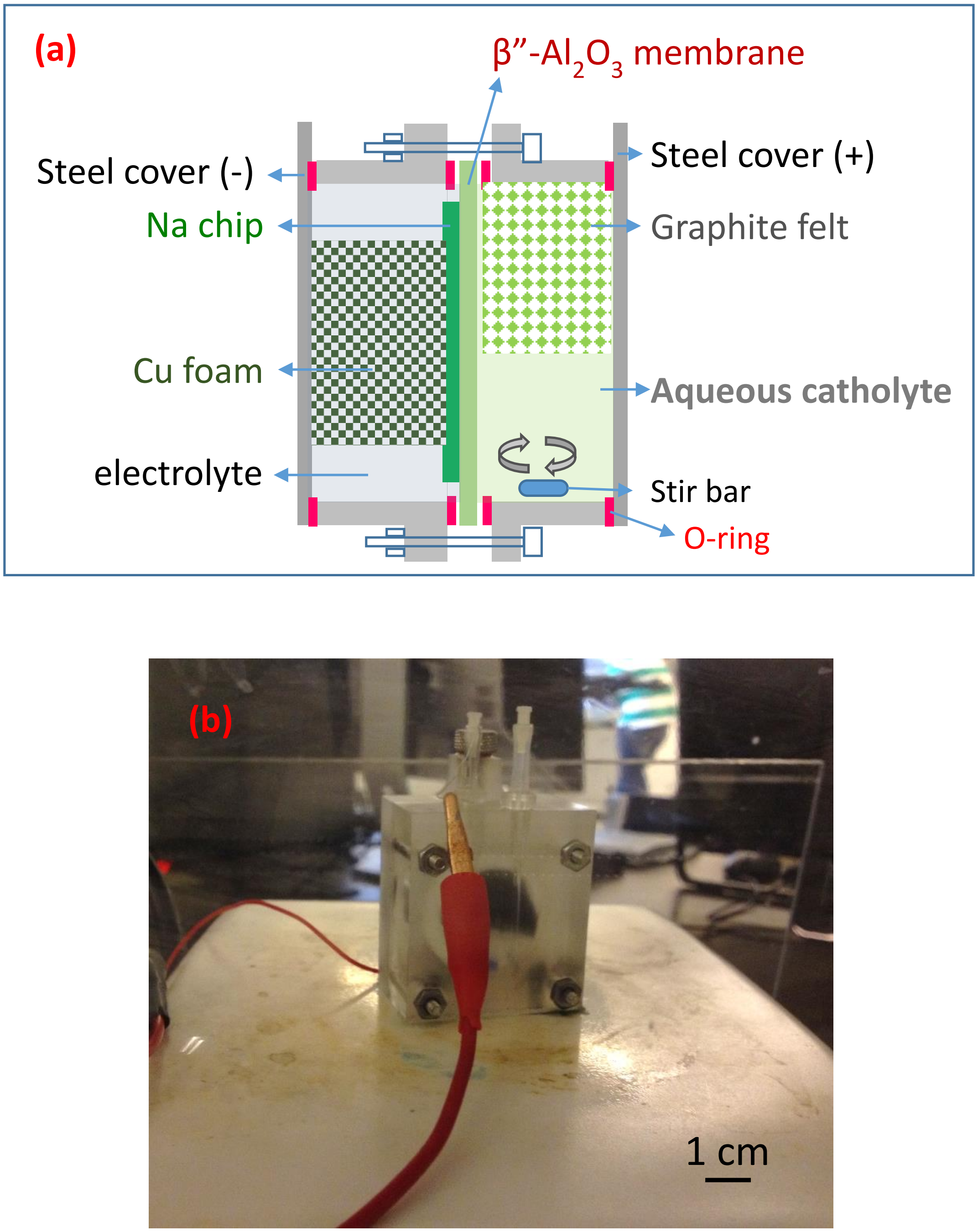

Figure S3: (a) Schematic of the cell structure for evaluating aqueous catholytes with more than one electron transfer redox reactions, and (b) a photo of the real cell setup.

S4: CV curves of aqueous electrolytes with and without addition of $BiCl_3$

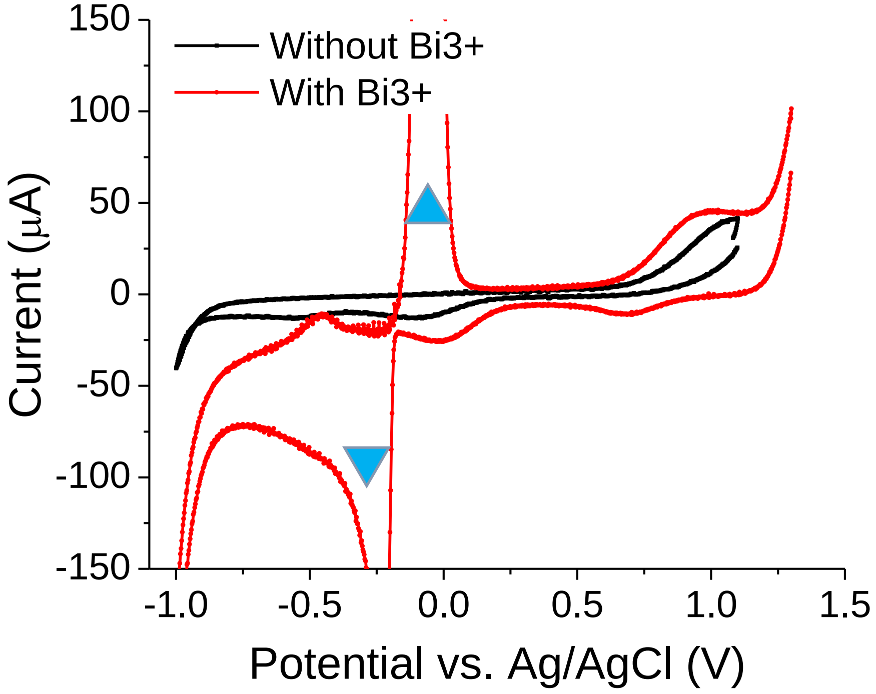

Figure S4: CV curves recorded at 250 mV/s for an aqueous catholyte with 0.01M $VOSO_4$ -0.05M Na_2SO_4 -1.5M HCl -0.002M $BiCl_3$, in which a glassy carbon (GC), Ag/AgCl, and Pt wire were used as the working, reference, and counter electrodes, respectively. The blue solid triangles (▼ and ▲) indicate Bi^{3+}/Bi redox reaction peaks. Clearly, the reversibility of all these V ion redox reactions are significantly improved after adding $BiCl_3$ into the catholyte.

S5: Nyquist plot for a full cell with a non-aqueous catholyte and Na-Cs anode

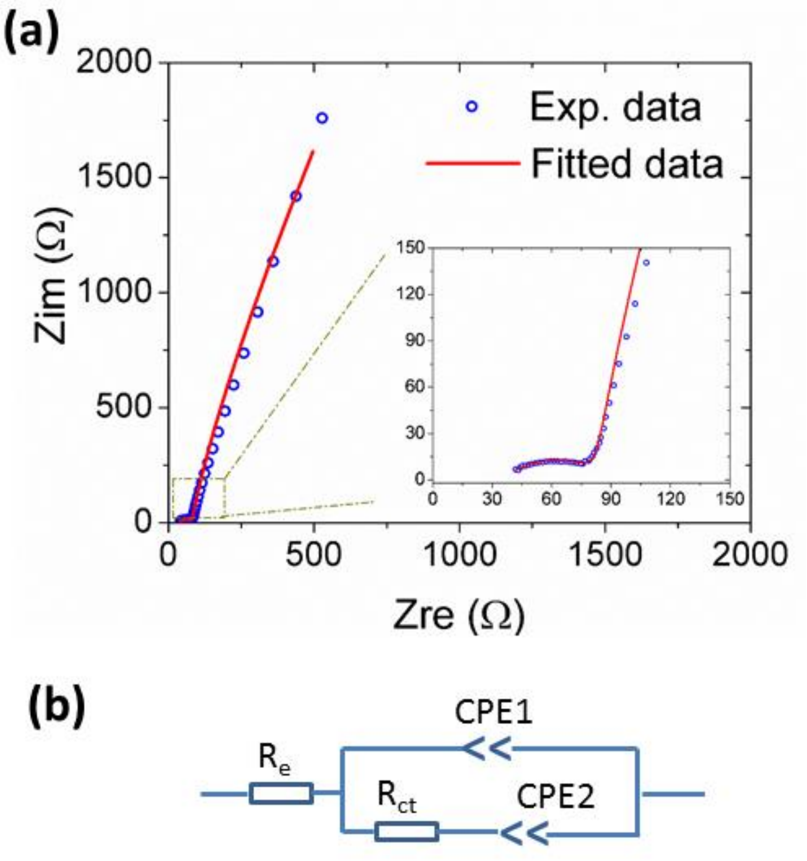

Figure S5: (a) Nyquist plot for a full cell made of a molten Na-Cs anode inside a moisture-sensitive β”-Al_2O_3 tube and the catholyte of 0.01M $V(acac)_3$ with 0.05M $NaPF_6$ in acetonitrile outside the tube before cycling, and (b) the equivalent circuit of the Nyquist plot based on model R(Q(QR)). The EIS measurements were performed at the open-circuit voltage of the cell in the frequency range of 10 kHz – 100 mHz with ac signal amplitude of 10 mV. The electronic resistance R_e (~ 35 Ω) and charge transfer resistance R_{ct} (~ 54 Ω) derived from the equivalent circuit are both quite small. The linear tail at low frequency is mainly due to the cathode acting as a double layer capacitor. It acts as a double layer capacitor because the initial OCV is between the $V^{3+/4+}$ and $V^{2+/3+}$ potentials.

S6: Nyquist plot of a moisture-resistant β"-Al_2O_3 membrane

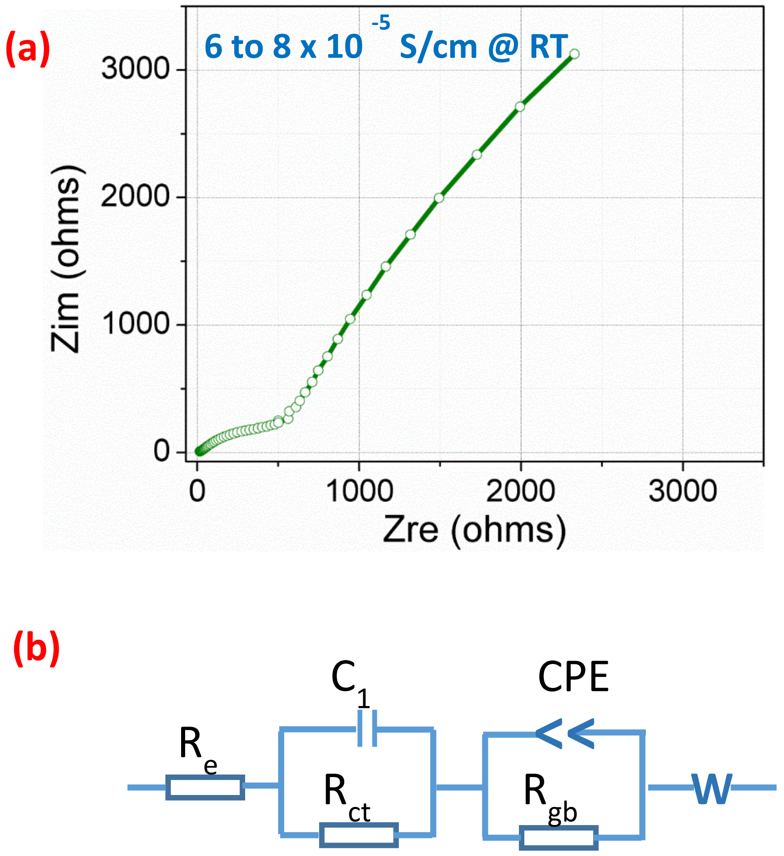

Figure S6: (a) Nyquist plot of the moisture-resistant β"-Al_2O_3 membrane obtained from Na_2SO_4 (1M aq.) | solid electrolyte | Na_2SO_4 (1M aq.) symmetric cell with the β"-Al_2O_3 membrane (diameter of 25.4 mm and thickness of 1.4 mm) as the solid electrolyte and using Cu foil strips as current collectors. The ionic conductivity, σ, was calculated by putting the grain boundary resistance (R_{gb}) in equation $\sigma = \frac{L}{Rgb \cdot A}$, in which R_{gb} are obtained by simulating via circuit R(RC)(RQ)W, shown in (b). The EIS measurements were performed at the open-circuit voltage of the cell in the frequency range of 100 kHz – 1 Hz with ac signal amplitude of 10 mV.

S7: XRD patterns of moisture-resistant β”-Al_2O_3 membranes

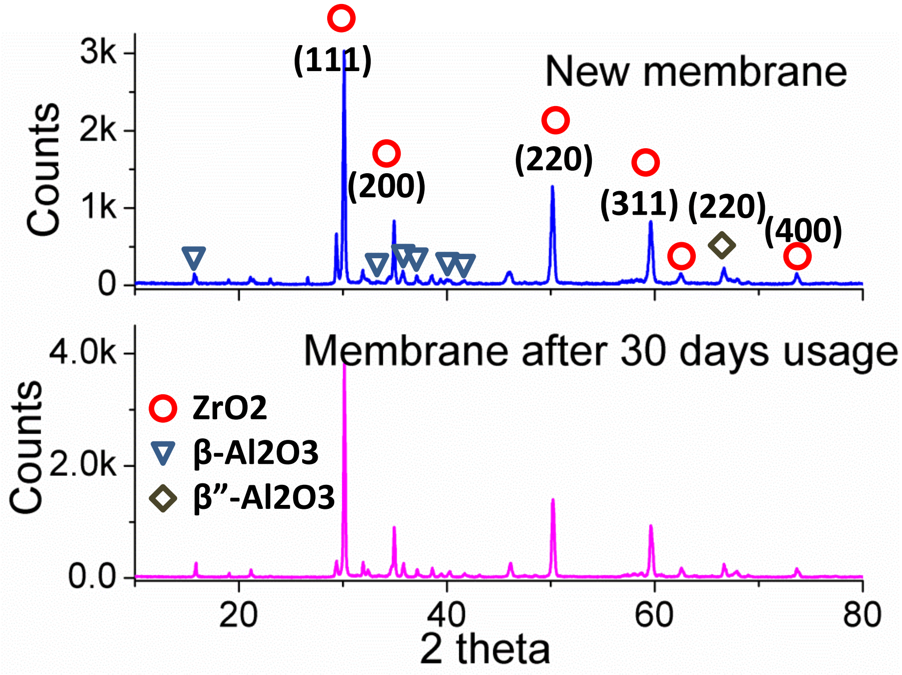

Figure S7: X-ray diffraction (XRD) patterns of the pristine/new solid electrolyte (blue, top) and the one used in Na|V batteries for at least 1 month (purple, bottom). The surface of used membrane was slightly polished. No obvious crystalline phase change could be observed.
